# Ketamine activates adult-born immature granule neurons to rapidly alleviate depression-like behaviors in mice

**DOI:** 10.1038/s41467-022-30386-5

**Published:** 2022-05-12

**Authors:** Radhika Rawat, Elif Tunc-Ozcan, Tammy L. McGuire, Chian-Yu Peng, John A. Kessler

**Affiliations:** Department of Neurology, Feinberg School of Medicine, Northwestern University, Chicago, IL 60611 USA

**Keywords:** Adult neurogenesis, Depression, Cellular neuroscience, Depression

## Abstract

Ketamine treatment decreases depressive symptoms within hours, but the mechanisms mediating these rapid antidepressant effects are unclear. Here, we demonstrate that activity of adult-born immature granule neurons (ABINs) in the mouse hippocampal dentate gyrus is both necessary and sufficient for the rapid antidepressant effects of ketamine. Ketamine treatment activates ABINs in parallel with its behavioral effects in both stressed and unstressed mice. Chemogenetic inhibition of ABIN activity blocks the antidepressant effects of ketamine, indicating that this activity is necessary for the behavioral effects. Conversely, chemogenetic activation of ABINs without any change in neuron numbers mimics both the cellular and the behavioral effects of ketamine, indicating that increased activity of ABINs is sufficient for rapid antidepressant effects. These findings thus identify a specific cell population that mediates the antidepressant actions of ketamine, indicating that ABINs can potentially be targeted to limit ketamine’s side effects while preserving its therapeutic efficacy.

## Abstract (web summary)

Rawat et al. demonstrate that activation of adult-born immature hippocampal neurons is necessary and sufficient for the acute antidepressant effects of low-dose ketamine in mice.

## Introduction

Major depressive disorder (MDD) is a leading contributor to disability worldwide, and its global disease burden continues to grow^1,2^. Antidepressant medications are limited by their efficacy and delayed therapeutic onset; most antidepressant medications require at least a week and up to 6 weeks for the onset of therapeutic effects^3^, and even then, nearly 1/3 of patients do not respond to treatment^2,4,5^. By contrast, even in antidepressant-resistant patients, a single dose of ketamine decreases depressive symptoms within hours and its effects last up to three weeks^6,7^. Although this effect is striking, ketamine use itself carries significant risks including addiction and other deleterious side effects^8–10^. These limitations highlight the need for mechanistic knowledge to inform new therapeutics with similar speed and magnitude of effects.

The mechanisms mediating the rapid antidepressant effect of ketamine are unclear. Ketamine is a non-competitive N-methyl-D-aspartate (NMDA) receptor antagonist^11^. However, while some other NMDA receptor antagonists produce rapid antidepressant effects^12–14^, many do not, indicating that additional mechanisms of action are likely involved^15–19^. Some effects have been linked to ketamine metabolites, such as hydroxynorketamine^20,21^, and to ketamine-induced alterations in signaling pathways, e.g., increased brain-derived neurotrophic factor (BDNF)^17,22^, eukaryotic elongation factor 2 kinase (eEF2K), mechanistic target of rapamycin (mTOR)^16,23^, and α-amino-3-hydroxy-5-methyl-4-isoxazole-propionic acid receptor (AMPAR) activation^23,24^. For example, ketamine-induced antidepressant effects require AMPAR-mediated activity in the rodent hippocampus and prefrontal cortex^13,23–25^, and inhibition of postsynaptic AMPAR by the small molecule NBQX, a selective, competitive AMPAR antagonist, prevents ketamine’s antidepressant effects^23,24,26^. Despite the many molecules, signaling pathways, and circuits involved, no specific, targetable cell population has yet been found to be necessary and sufficient to mediate ketamine’s effects.

Ketamine exerts effects on many different brain regions including the hippocampus^12,18,25,27–29^. Multiple lines of evidence suggest that hippocampal dysfunction is involved in MDD^30^, and the volume of the hippocampus is significantly decreased in patients with MDD^31^. Conventional antidepressants increase adult hippocampal neurogenesis, i.e., development of new granule neurons, possibly reversing the cellular changes induced by depression^32–34^, and inhibiting neurogenesis blocks the behavioral effects of these antidepressants in rodent and nonhuman primate models^32,35–37^. It takes several weeks for stem/progenitor cells to generate new granule neurons that integrate into the hippocampal circuitry, and the delay in the onset of action of most antidepressants has been ascribed to the time necessary to generate new neurons^38–40^. However, neurogenesis does not have to be altered for the acute effect of ketamine to be realized^41^. Acute activation of adult-born immature granule neurons (ABINs) in the dentate gyrus (DG) of the hippocampus can induce a rapid antidepressant effect in mice^40^, suggesting a potential cellular mechanism underlying the acute effects of ketamine. In the present study, we used a chemogenetic approach in combination with a transgenic mouse model to establish that activation of ABINs is both necessary and sufficient to produce the rapid behavioral effects of ketamine.

## Results

### Ketamine treatment increases the activity of immature DG neurons

**Fig. 1:**
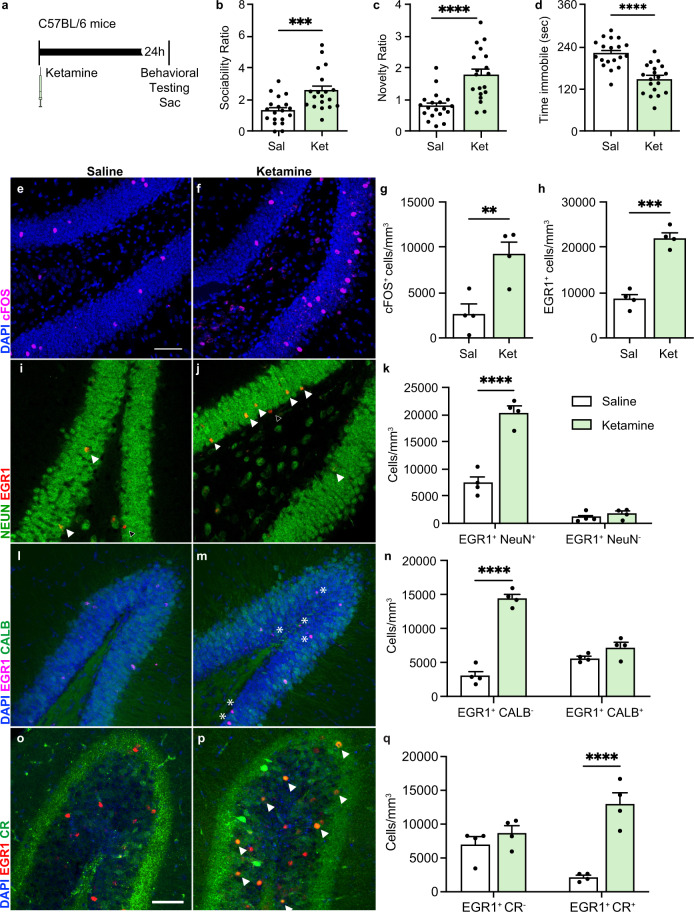
Ketamine treatment increases the activity of immature DG neurons. **a** Experimental timeline. Saline or ketamine was administered by intraperitoneal (i.p.) injection to wild-type C57BL/6 mice 24 h prior to behavioral testing. Immediately following testing, mice were euthanized for sample collection. **b** Sociability Ratio. Student’s *t*-test: saline (*n* = 19) vs ketamine (*n* = 19) ***p = 0.0009. **c** Novelty Ratio. Saline (*n* = 19) vs ketamine (*n* = 19) ****p < 0.0001 by Welch’s *t*-test. **d** Time immobile in seconds. Student’s *t*-test: Saline (*n* = 19) vs ketamine (*n* = 19) ****p < 0.0001). **e**–**g** Representative immunohistochemical (IHC) staining of cFOS^+^ (magenta) cells in the dentate gyrus (DG) and quantification of cFOS^+^ cells/mm^3^ (*n* = 4 mice/group) **p = 0.0096 by Student’s *t*-test. **h** Quantification of EGR1^+^ cells/mm^3^. saline (*n* = 4) vs ketamine (*n* = 4) ***p < 0.0001 by Student’s *t*-test. **i**, **j** Representative IHC staining of NeuN^+^ (green), Egr1^+^ (red, outlined arrow), and double-positive (yellow, solid white arrow) cells in the DG. **k** Quantification of EGR1^+^NeuN^+^ cells and EGR1^+^NeuN^−^ cells in saline-treated and ketamine-treated mouse DGs (*n* = 4 mice/group). Two-way ANOVA: NeuN *F*_1, 12_ = 205.0 ****p < 0.0001, ketamine *F*_1, 12_ = 60.95 ****p < 0.0001; NeuN*ketamine *F*_1,12_ = 50.20 ****p < 0.0001; Tukey’s post hoc test: saline NeuN^+^ vs saline NeuN^−^ **p = 0.0013, saline NeuN^+^ vs ketamine NeuN^+^ ****p < 0.0001, saline NeuN^+^ vs ketamine NeuN^−^ **p = 0.0029, saline NeuN^−^ vs ketamine NeuN^+^ ****p < 0.0001, saline NeuN^−^ vs ketamine NeuN^−^ not significant (ns) *p* = 0.955, ketamine NeuN^+^ vs ketamine NeuN^−^ ****p < 0.0001). **l**, **m** Representative IHC staining of EGR1^+^ (magenta, white asterisk), Calbindin (Calb)^+^ (green), and double-positive (pink) cells in the DG. **n** Quantification of EGR1^+^Calb^−^ cells and EGR1^+^Calb^+^ cells in saline-treated and ketamine-treated mouse DGs (*n* = 4 mice/group). Two-way ANOVA: Calb *F*_1, 12_ = 15.08 **p = 0.0022, ketamine *F*_1, 12_ = 115.1 ****p < 0.001, Calb*ketamine *F*_1, 12_ = 66.96 ****p < 0.0001; Tukey’s post hoc test: saline Calb^−^ vs ketamine Calb^−^ ****p < 0.0001, saline Calb^−^ vs saline Calb^+^ *p = 0.0442, saline Calb^−^ vs Calb^+^ ketamine **p = 0.002, ketamine Calb^−^ vs saline Calb^+^ ****p < 0.0001, ketamine Calb^−^ vs ketamine Calb^+^ ****p < 0.0001, saline Calb^+^ vs ketamine Calb^+^ ns *p* = 0.3192. **o**, **p** Representative IHC staining of EGR1^+^ (red) and Calretinin (CR)^+^ (green) cells in the DG. White arrows depict example double-positive cells. **q** Quantification of EGR1^+^CR^−^ cells and EGR1^+^CR^+^ cells in saline-treated and ketamine-treated mouse DGs (*n* = 4 mice/group). Two-way RM ANOVA: CR *F*_1,6_ = 0.1254 ns *p* = 0.7353; ketamine *F*_1,6_ = 19.10 **p = 0.0047; Sidak’s multiple comparisons test: saline CR^−^ vs ketamine CR^−^ ns *p* = 0.5193, saline CR^+^ vs ketamine CR^+^ ****p < 0.0001. Data were presented as means ± s.e.m. Each replicate is a different mouse. Source data and complete statistics are provided as a source data file. Scale bars 50 μm.

We first examined the acute behavioral effects of ketamine treatment in wild-type C57BL/6 mice. The social interaction (SIT), social novelty (SNT), and tail suspension (TST) tests have been used widely to assess hippocampus-dependent anxiety- and depression-like behavior and social memory^40,42–44^ and were used throughout this study. Consistent with prior reports, we observed significant effects on affective behavior, 24 h after a single subanesthetic dose of ketamine (3 mg/kg intraperitoneal (i.p.) injection) (Fig. 1)^26^. We tested both male and female mice, and no behavioral differences were observed between the sexes (Supplementary Fig. 1a–d; source data and complete statistics are provided as a source data file); therefore their data were analyzed together (Fig. 1a–d). Compared to saline-treated animals, ketamine-treated mice displayed increased sociability in the SIT (*p* < 0.001), increased preference for social novelty in the SNT (*p* < 0.001) and decreased immobility in the TST (*p* < 0.0001) (Fig. 1a–d). Ketamine had no effect on overall locomotor activity measured by the total distance traveled in the open field test (Supplementary Fig. 1e).

Preference for social interaction, novelty, and other affective behaviors are regulated by the hippocampus and dentate gyrus (DG)^40,42–51^. We, therefore, asked whether ketamine treatment alters DG neuronal activity alongside the observed changes in behavior. The immediate-early genes, cFOS and early growth response 1 (EGR1) are both upregulated by neuronal activity and are used as proxies of neuronal activity^52^. Mice from each group were randomly selected for immunohistochemical analysis. A single dose of ketamine significantly increased the number of cFOS^+^ (*p* < 0.01) and EGR1^+^ (*p* < 0.001) activated neurons in the DG (Fig. 1e–h). To better define the identity of the neuronal populations activated by ketamine, we analyzed the cell types that expressed EGR1. As postmitotic neurons mature, they sequentially express neuronal markers, from NeuroD1 and doublecortin (DCX) in neural progenitors, to NeuN in neurons, and then to Calbindin which is expressed in mature but not immature granule neurons^53^. Nearly all active (EGR1^+^) cells expressed NeuN, a common neuronal marker^54^ (Fig. 1i–k), whereas virtually no active cells (EGR1^+^ or cFOS^+^) expressed the neuronal progenitor cell marker, NeuroD1, or the neuroblast/immature neuron marker, DCX (Supplementary Fig. 1f, g). Furthermore, ketamine treatment significantly increased the number of EGR1-positive granule cells that did not express Calb (DAPI^+^EGR1^+^Calb^–^) while the number of EGR1-expressing cells that also expressed Calb (DAPI^+^EGR1^+^Calb^+^) was unchanged (Fig. 1l–n), indicating that ketamine primarily activated immature neurons in the DG alongside its acute effects on behavioral phenotypes. To more specifically identify the lineage stage affected by ketamine, we analyzed the expression of EGR1 by Calretinin^+^ cells. Calretinin (CR) is transiently expressed by immature postmitotic neurons before they mature and express Calb^55^. Ketamine treatment significantly increased the number of EGR1^+^CR^+^ but not EGR1^+^CR^–^ cells (Fig. 1o–q). Thus, ketamine activates immature neurons at a stage after NeuroD and DCX expression but before the cells mature into Calb^+^ mature granule neurons.

### Silencing adult-born immature granule neurons (ABINs) blocks the acute behavioral effects of ketamine as well as its ability to rescue stress-induced behavioral phenotypes

**Fig. 2:**
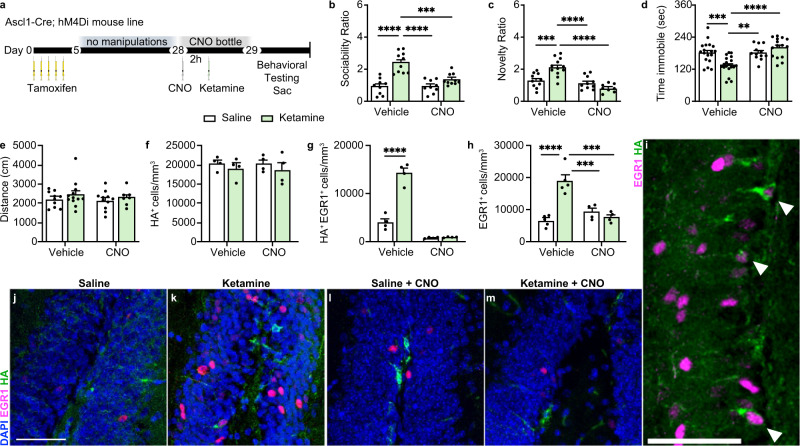
Selectively silencing adult-born immature granule neurons (ABINs) blocks the acute effects of ketamine. **a** Experimental timeline. Ascl1-Cre;hM4Di mice received once-daily tamoxifen injections for 5 days. Three weeks after the first tamoxifen dose, CNO or vehicle was administered by intraperitoneal injection (i.p.) and 2 h later, mice were given an i.p. injection of saline or ketamine. Either CNO or vehicle was provided ad libitum overnight. Twenty-four hours after saline or ketamine treatment, mice underwent behavioral testing and euthanasia immediately afterward for sample collection. **b** Sociability Ratio. Two-way ANOVA: CNO*ketamine *F*_1, 34_ = 11.09 **p = 0.0021, CNO *F*_1,34_ = 11.12 **p = 0.0021, ketamine *F*_1,34_ = 36.45 ****p < 0.0001; Tukey’s post hoc test: vehicle + saline (*n* = 9) vs vehicle + ketamine (*n* = 10) ****p < 0.0001, vehicle + ketamine vs CNO + saline (*n* = 9) ****p < 0.0001, vehicle + ketamine vs CNO + ketamine (*n* = 10) ***p = 0.0002, vehicle + saline vs CNO + saline ns *p* > 0.9999, vehicle + saline vs CNO + ketamine ns *p* = 0.2425, CNO + saline vs CNO + ketamine ns *p* = 0.2413. **c** Novelty Ratio. Two-way ANOVA: CNO*ketamine *F*_1, 36_ = 16.62 ***p = 0.0002, CNO *F*_1, 36_ = 25.63 ****p < 0.0001, ketamine *F*_1, 36_ = 3.047 ns *p* = 0.0894; Tukey’s post hoc test: vehicle+saline (*n* = 10) vs. vehicle + ketamine (*n* = 12) ***p = 0.0006, vehicle + ketamine vs. CNO + saline (*n* = 10) ****p < 0.0001, vehicle + ketamine vs. CNO + ketamine (*n* = 8) ****p < 0.0001, vehicle + saline vs. CNO + saline ns *p* = 0.8948,vehicle + saline vs. CNO + ketamine ns *p* = 0.1334, CNO + saline vs. CNO + ketamine ns *p* = 0.4078. **d** Time immobile in seconds. Two-way ANOVA: CNO *F*_1, 60_ = 12.93, ***p = 0.0007; ketamine *F*_1,60_ = ns *p* = 0.1060; CNO*ketamine *F*_1,60_ = 13.65, ***p = 0.0005. Tukey’s post hoc test: vehicle + saline(*n* = 18) vs vehicle + ketamine (*n* = 19) ***p = 0.0007; vehicle + saline vs CNO + saline (*n* = 12) ns *p* = 0.999; vehicle + saline vs CNO + ketamine (*n* = 15) ns *p* = 0.4919; vehicle + ketamine vs CNO + saline **p = 0.0034; vehicle + ketamine vs CNO + ketamine ****p < 0.0001; CNO + saline vs CNO + ketamine ns *p* = 0.53. **e** Distance traveled in centimeters (vehicle + saline *n* = 10, vehicle + ketamine *n* = 12, CNO + saline *n* = 11, CNO + ketamine *n* = 7). Two-way ANOVA ns. **f** Quantification of HA^+^ cells/mm^3^ in the DG (*n* = 4 mice/group). Two-way ANOVA ns. **g** Quantification of HA^+^EGR1^+^ cells/mm^3^. Two-way ANOVA: CNO *F*_1,12_ = 157.3 ***p < 0.0001, ketamine *F*_1,12_ = 61.26 ****p < 0.0001, CNO*ketamine *F*_1,12_ = 58.56 ****p = 0.0001; Tukey’s post hoc test: vehicle + saline vs vehicle + ketamine ****p < 0.0001, vehicle + saline vs CNO + saline *p = 0.0213, vehicle + saline vs CNO + ketamine *p = 0.0265, vehicle + ketamine vs CNO + saline ****p < 0.0001, vehicle + ketamine vs CNO + ketamine ****p < 0.0001, CNO + saline vs CNO + ketamine ns *p* = 0.9993. **h** Quantification of EGR1^+^ cells/mm^3^ (*n* = 4 mice/group). Two-way ANOVA: CNO*ketamine *F*_1,14_ = 28.83 *p* < 0.0001, CNO *F*_1, 14_ = 11.09 **p = 0.0050, ketamine *F*_1, 14_ = 17.03 **p = 0.0010; Tukey’s post hoc test: vehicle + saline vs vehicle + ketamine ****p < 0.0001, vehicle + ketamine vs CNO + saline ***p = 0.0006, vehicle + ketamine vs CNO + ketamine ***p = 0.0001, CNO + saline vs CNO + ketamine ns *p* = 0.8376, vehicle + saline vs CNO + saline ns *p* = 0.4957, vehicle + saline vs CNO + ketamine ns *p* = 0.9414. **i**–**m** Representative IHC of EGR1^+^ (magenta, nuclear) and HA^+^ (green, cytoplasmic) colocalization. White arrows indicate colocalized cells. Data were presented as means ± s.e.m. Each replicate is a different mouse. Source data and complete statistics are provided as a source data file. Scale bars 50 μm.

Activation of ABINs induces a potent, near-immediate positive behavioral effect in mice^40^. We thus hypothesized that the increased number of active ABINs observed in the DG may mediate the rapid behavioral effects induced by ketamine. To test this hypothesis, we used a chemogenetic approach combined with a transgenic mouse model^40,56^ to selectively inhibit the activity of ABINs in the DG. We used the Ascl1-Cre;hM4Di mouse model described previously^40^, hereafter called “hM4Di,” to induce specific and inducible inhibition of ABIN activity. The designer receptor exclusively activated by designer drugs (DREADD), hM4Di, is a synthetic variant of the human muscarinic receptor. hM4Di interacts specifically and with high efficacy with the exogenous ligand clozapine-N-oxide (CNO), and it initiates the canonical inhibitory Gi pathway thereby inhibiting cellular activity^56–58^. Expression of hM4Di in the absence of CNO, or exposure of wild-type neurons to CNO, does not affect neuronal activity; the hM4Di receptor and CNO are inert elements until combined^40,56,58^. Furthermore, there are no observed morphological effects before or after tamoxifen and CNO administration^40^. Upon administration of tamoxifen (TAM), the hM4Di mouse expresses the HA-tagged hM4Di in Type-2 transient amplifying progenitors (NPCs) and their progeny, as well as in a small subset of Type 1 neural stem cells (NSCs)^59^. Three weeks after TAM treatment, the tagged Type-2 cells have matured, and HA-tagged hM4Di is expressed almost exclusively in NeuN^+^Calb^–^ immature neurons^59^, at which point, hM4Di mice were given either CNO (to block activation of ABINs) or vehicle, and, 2 h later, ketamine or saline. Twenty-four hours later these mice underwent behavioral testing (Fig. 2a). In vehicle-treated mice, ketamine administration resulted in a significantly increased social interaction ratio (*p* < 0.0001) (Fig. 2b), increased preference for novelty (*p* < 0.001) (Fig. 2c), and decreased time immobile (*p* < 0.001) (Fig. 2d). CNO administration prevented these behavioral changes in ketamine-treated mice but had no effect in saline-treated mice compared to the control (vehicle + saline) group. No differences in locomotion among groups were observed, as measured by the total distance traveled during the open field test (Fig. 2e).

Ketamine treatment did not affect the total number of ABINs (HA^+^ cells) (Fig. 2f) but did significantly increase the number of activated ABINs (EGR1^+^HA^+^) (*p* < 0.0001) (Fig. 2g) as well as the overall number of activated (EGR1^+^) cells (*p* < 0.0001) (Fig. 2h–m). CNO treatment virtually abolished EGR1 expression in HA^+^ cells (Fig. 2g) and prevented the increase in overall numbers of EGR1^+^ cells (Fig. 2h) after ketamine treatment. CNO administration to saline-treated mice did not alter the total number of active cells compared to vehicle + saline treatment (Fig. 2h). These results demonstrate that specifically silencing DG ABINs in hM4Di mice blocks the acute behavioral effects of ketamine.

**Fig. 3:**
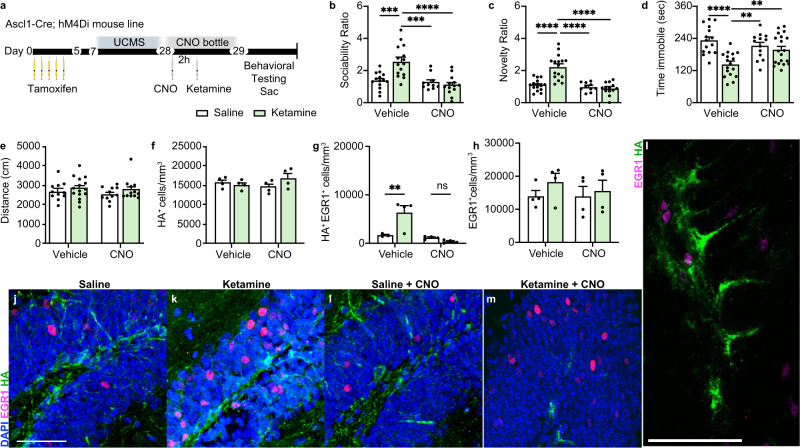
Selectively silencing ABINs blocks ketamine’s ability to rescue stress-induced behavioral phenotypes. **a** Experimental timeline. Ascl1-Cre;hM4Di mice received once-daily tamoxifen injections for 5 days. On day 7, a 3-week paradigm of unpredictable mild chronic stress (UCMS) was started. CNO or vehicle then was administered by intraperitoneal injection (i.p.) and 2 h later, mice were given an i.p. injection of saline or ketamine. Either CNO or vehicle was provided ad libitum *overnight*. 24 h after saline or ketamine treatment, mice underwent behavioral testing and euthanasia immediately afterward for sample collection. **b** Sociability Ratio. Two-way ANOVA: Interaction *F*_1, 48_ = 12.76 ***p = 0.0008, CNO *F*_1, 48_ = 16.88 ***p = 0.0002, ketamine *F*_1, 48_ = 6.716 *p = 0.0126; Tukey’s post hoc test: vehicle + saline (*n* = 14) vs vehicle + ketamine (*n* = 15) ***p = 0.0001, vehicle + ketamine vs CNO + saline (*n* = 10) ***p = 0.0002, vehicle + ketamine vs CNO + ketamine (*n* = 13) ****p < 0.0001, vehicle + saline vs CNO + saline ns *p* = 0.9833, vehicle + saline vs CNO + ketamine ns *p* = 0.6882, CNO + saline vs CNO + ketamine ns *p* = 0.9137. **c** Novelty Ratio. Two-way ANOVA: Interaction *F*_1, 50_ = 19.73 ****p < 0.0001, CNO *F*_1,50_ = 28.00 ****p < 0.0001, ketamine ***F_1, 50_ = 13.81 *p* = 0.0005; Tukey’s post hoc test: vehicle + saline (*n* = 15) vs vehicle + ketamine (*n* = 16) ****p < 0.0001, vehicle + saline vs CNO + saline (*n* = 10) ns *p* = 0.9389, vehicle + saline vs CNO + ketamine (*n* = 13) ns *p* = 0.6606, vehicle + ketamine vs CNO + saline ****p < 0.0001, vehicle + ketamine vs CNO + ketamine ****p < 0.0001, CNO + saline vs CNO + ketamine ns *p* = 0.9637. **d** Time immobile in seconds. Two-way ANOVA: Interaction *F*_1, 60_ = 9.515 **p = 0.0031, CNO *F*_1, 60_ = 1.813 *p* = 0.1832, ketamine *F*_1, 60_ = 17.45 ****p < 0.0001; Tukey’s post hoc test: vehicle + saline (*n* = 15) vs vehicle + ketamine (*n* = 17) ****p < 0.0001, vehicle + ketamine vs CNO + saline (*n* = 13) **p = 0.002, vehicle + ketamine vs CNO + ketamine (*n* = 19) **p = 0.0074, vehicle + saline vs CNO + saline ns *p* = 0.6552, vehicle + saline vs CNO + ketamine ns *p* = 0.1749, CNO + saline vs CNO + ketamine ns *p* = 0.8692. **e** Distance traveled in centimeters (vehicle + saline n = 11, vehicle+ketamine *n* = 15, CNO + saline *n* = 11, CNO + ketamine *n* = 12). Two-way ANOVA: ns. **f** Quantification of HA^+^ cells/mm^3^ in the DG (*n* = 4 mice/group). Two-way ANOVA: ns. **g** Quantification of HA^+^EGR1^+^ cells/mm^3^ (*n* = 4 mice/group). Two-way ANOVA: Interaction *F*_1, 11_ = 11.65 **p = 0.0058, CNO *F*_1, 11_ = 16.35 **p = 0.0019, Antidepressant *F*_1, 11_ = 5.861 *p = 0.0339; Tukey’s post hoc test: vehicle + saline vs vehicle + ketamine **p = 0.01, vehicle + saline vs CNO + saline ns *p* = 0.9721, vehicle + saline vs CNO + ketamine ns *p* = 0.6936, vehicle + ketamine vs CNO + saline **p = 0.0028, vehicle + ketamine vs CNO + ketamine ***p = 0.0009, CNO + saline vs CNO + ketamine ns *p* = 0.883. **h** EGR1^+^ cells/mm^3^ (*n* = 4 mice/group). Two-way ANOVA: ns. **i**–**m** Representative IHC of EGR1^+^ (magenta) and HA^+^ (green) staining. Data were presented as means ± s.e.m and were analyzed by *t*-test or ANOVA and Tukey’s post hoc test. Each replicate is a different mouse. Source data and complete statistics are provided as a source data file. Scale bars 50 μm.

We next investigated the role of ABIN activity in the antidepressant effect of ketamine in an animal model of depression. Unpredictable chronic mild stress (UCMS) is a widely used, well-validated, and reliable behavioral model of depression that induces a robust and long-lasting depression-like phenotype in mice^40,60–67^. Because there are reports that the effects of ketamine differ based on stress exposure^68,69^, we tested whether ABIN activation is also crucial for the ability of ketamine to reverse the effects of stress in mice exposed to UCMS. After TAM administration, hM4Di mice underwent UCMS for 3 weeks using our previously established protocol^40^ (and see Methods and Supplementary Tables 2,3) followed by treatment with vehicle or CNO and either saline or ketamine (Fig. 3a), similar to the above experiments with unstressed mice. In vehicle-treated stressed mice, ketamine administration increased sociability (*p* < 0.001) (Fig. 3b) and preference for novelty (*p* < 0.0001) (Fig. 3c), and decreased immobility (*p* < 0.0001) (Fig. 3d). Stressed mice displayed significantly more depression-like behavior than stress-naïve mice, and ketamine treatment increased mobility for both (Supplementary Fig. 2a). CNO administration prevented these behavioral changes in ketamine-treated UCMS mice but had no effect in saline-treated mice (Fig. 3a–d). There was no change in locomotor behavior among groups (Fig. 3e). Thus, the acute antidepressant-like effects of ketamine require immature DG neuron activity both in the presence and absence of chronic stress.

As in the unstressed experiment, ketamine treatment did not alter the total number of HA^+^ cells in UCMS exposed mice (Fig. 3f), and ketamine administration to mice exposed to UCMS significantly increased the number of activated ABINs (EGR1^+^HA^+^) (*p* < 0.01) (Fig. 3g). The total number of EGR1^+^ cells was not significantly different among groups (Fig. 3h). Overall, CNO treatment blocked the increase in ketamine-induced ABIN activity (Fig. 3g, i–m).

The increase in EGR1^+^HA^+^ cells occurred primarily in the ventral hippocampus (Supplementary Fig. 2b), consistent with reports that the acute effects of ketamine are observed primarily within the ventral hippocampus and alter ventral hippocampus-associated affective behavior^70^ rather than on dorsal hippocampus-associated cognitive behaviors^71^. In addition, the number of DCX^+^ cells was not altered 24 h after ketamine treatment (Supplementary Fig. 2c). However, mice exposed to the 3-week unpredictable chronic stress paradigm had fewer HA^+^ cells/mm^3^ than the stress-naïve mice (Supplementary Fig. 2d), suggesting that neurogenesis decreased during exposure to chronic stress, consistent with previous reports^40,72^. Overall, these results demonstrate that the acute effects of ketamine did not require an increase in neurogenesis but rather an increase in the activity of ABINs in the DG.

### Ketamine’s effects on adult-born immature granule neurons (ABINs) and behavior require AMPA Receptors

**Fig. 4:**
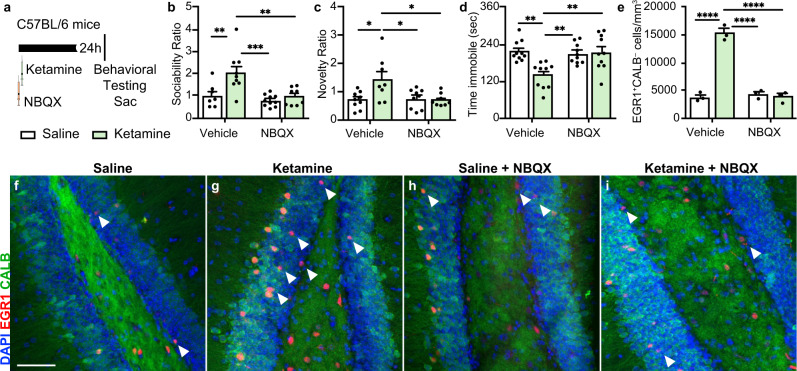
Ketamine’s effects on ABINs and mouse behavior require AMPA receptors. **a** Experimental timeline. Wild-type C57BL/6 mice received two injections 24 h prior to behavioral testing: either saline or ketamine by intraperitoneal (i.p.) injection and either NBQX or vehicle (vehicle) by subcutaneous injection. Immediately following testing, mice were euthanized for sample collection. **b** Sociability Ratio. Two-way ANOVA: Interaction *F*_1, 30_ = 4.514 *p = 0.0420, NBQX *F*_1, 30_ = 10.98 **p = 0.0024, ketamine *F*_1, 30_ = 9.107 **p = 0.0052; Tukey’s post hoc test: vehicle + saline vs vehicle + ketamine **p = 0.007, vehicle + ketamine (*n* = 9) vs NBQX + saline (*n* = 10) ***p = 0.0003, vehicle + ketamine vs NBQX + ketamine (*n* = 8) **p = 0.0029, NBQX + saline vs NBQX + ketamine ns *p* = 0.9142, vehicle + saline (*n* = 7) vs NBQX + saline ns *p* = 0.8377, vehicle + saline vs NBQX + ketamine ns *p* = 0.9972. **c** Novelty Ratio. Two-way ANOVA: Interaction *F*_1, 30_ = 6.101 *p = 0.0194, NBQX *F*_1, 30_ = 4.520 *p = 0.0418, ketamine *F*_1, 30_ = 4.717 *p = 0.0379; Tukey’s post hoc test: vehicle + saline (*n* = 9) vs vehicle + ketamine (*n* = 8) *p = 0.0132, vehicle + ketamine vs NBQX + saline (*n* = 9) *p = 0.0239, vehicle + ketamine vs NBQX + ketamine (*n* = 8) *p = 0.0179, NBQX + saline vs NBQX + ketamine ns *p* = 0.9966, vehicle + saline vs NBQX + saline ns *p* = 0.9943, vehicle + saline vs NBQX + ketamine ns >0.9999. **d** Time immobile in seconds. Two-way ANOVA: Interaction *F*_1, 36_ = 9.580 **p = 0.0038, CNO *F*_1,36_ = 6.074 *p = 0.0186, ketamine *F*_1, 36_ = 7.328 *p = 0.0103; Tukey’s post hoc test: vehicle + saline (*n* = 10) vs vehicle + ketamine (*n* = 10) **p = 0.0012, vehicle + ketamine vs NBQX + saline (*n* = 10) **p = 0.0043, vehicle + ketamine vs NBQX + ketamine (*n* = 10) **p = 0.002, vehicle + saline vs NBQX + saline ns *p* = 0.97, vehicle + saline vs NBQX + ketamine ns *p* = 0.9982, NBQX + saline vs NBQX + ketamine ns *p* = 0.9927. **e** Quantification of EGR1^+^Calb^–^ single-positive cells (n = 3 mice/group). Two-way ANOVA: Interaction *F*_1, 8_ = 106.7 ****p < 0.0001, NBQX *F*_1, 8_ = 88.58 ****p < 0.0001, ketamine *F*_1, 8_ = 88.93 ****p < 0.0001; Tukey’s post hoc test: vehicle + saline vs vehicle + ketamine ****p < 0.0001, vehicle + saline vs NBQX + saline ns *p* = 0.9128, vehicle + saline vs NBQX + ketamine ns >0.9999, vehicle + ketamine vs NBQX + saline ****p < 0.0001, vehicle + ketamine vs NBQX + ketamine ****p < 0.0001, NBQX + saline vs NBQX + ketamine ns *p* = 0.9173. **f**–**i** Representative IHC staining of EGR1^+^ (red) single-positive cells (indicated by a white arrow), Calb^+^ (green) cells, and double-positive (yellow) cells in the DG. Data are presented as means ± s.e.m. Each replicate is a different mouse. Source data and complete statistics are provided as a source data file. Scale bars 50 μm.

AMPAR activation occurs in response to ketamine treatment of mice, and prior work has demonstrated that AMPAR inhibition abolishes ketamine’s acute effects^23,24,29^. To examine how our observations about ABIN activity may integrate into current hypotheses of ketamine action, we tested whether inhibiting AMPA receptors prevents the activation of ABINs and the behavioral effects of ketamine. Wild-type mice treated with ketamine or saline were also treated with either the AMPAR antagonist (NBQX) or vehicle (Fig. 4a). Consistent with numerous reports^23,24,73^, treatment with NBQX prevented the effects of ketamine on behavior in the SIT, SNT, and TST (4b-d). NBQX also prevented the ketamine-induced increase in EGR1^+^Calb^–^ cells (Fig. 4e–i). As before, the EGR1^+^ cells were predominantly NeuN^+^, confirming their neuronal identity. These results indicate that the activation of ABINs, which we found was necessary for the acute behavioral effects of ketamine, depends upon AMPAR activation.

### Activation of adult-born immature granule neurons (ABINs) is sufficient to induce ketamine-like behavioral effects

**Fig. 5:**
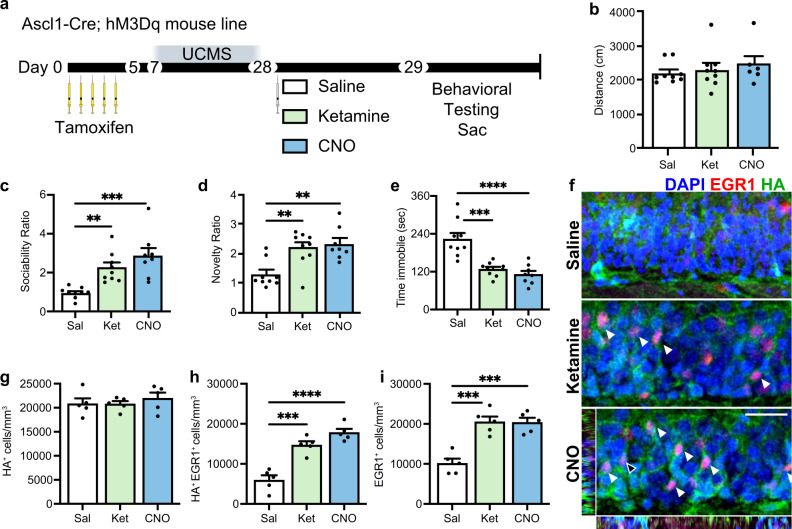
Activation of ABINs is sufficient to induce ketamine-like behavioral effects. **a** Experimental timeline. Ascl1-Cre;hM3Dq mice received once-daily tamoxifen injections for 5 days. On day 7, a 3-week paradigm of unpredictable mild chronic stress (UCMS) was started. On day 28, saline, ketamine, or CNO was administered by intraperitoneal injection (i.p.) and 24 h later, mice underwent behavioral testing and euthanasia immediately afterward for sample collection. **b** Distance traveled in centimeters (saline *n* = 9, ketamine *n* = 9, CNO *n* = 6). ANOVA: ns. **c** Sociability Ratio. ANOVA: Treatment *F*_2, 23_ = 12.53 ***p = 0.0002; Tukey’s post hoc test: saline (*n* = 9) vs ketamine (*n* = 9) **p = 0.0075, saline vs CNO (*n* = 8) ***p = 0.0002, ketamine vs CNO ns *p* = 0.2535. **d** Novelty Ratio. ANOVA: Treatment F_2, 23_ = 9.602 ***p = 0.0009; Tukey’s post hoc test: saline (*n* = 9) vs ketamine (*n* = 9) **p = 0.0045, saline vs CNO (*n* = 8) **p = 0.0018, ketamine vs CNO ns *p* = 0.8751. **e** Time immobile in seconds. ANOVA: Treatment *F*_2, 23_ = 19.19 ****p < 0.0001; Tukey’s post hoc test: saline (*n* = 9) vs ketamine (*n* = 9) ***p = 0.0001, saline vs CNO (*n* = 8) ****p < 0.0001, ketamine vs CNO ns *p* = 0.6859. **f** Representative IHC of EGR1^+^ (red) and HA^+^ (green) staining. White arrows depict colocalization. Orthogonal views represent the coordinates of the cell marked with a black arrow. **g** Quantification of HA^+^ cells/mm^3^ in the DG (*n* = 5 mice/group). ANOVA: ns. **h** Quantification of HA^+^EGR1^+^ cells/mm^3^ (*n* = 5 mice/group). ANOVA: Treatment *F*_2, 12_ = 35.96 ****p < 0.0001; Tukey’s post hoc test: saline vs ketamine ***p = 0.0002, saline vs CNO, ****p < 0.0001, ketamine vs CNO ns *p* = 0.1202. **i** EGR1^+^ cells/mm^3^ (*n* = 5 mice/group). ANOVA: Treatment *F*_2, 12_ = 23.20 ****p < 0.0001; Tukey’s post hoc test: saline vs ketamine ***p = 0.0002, saline vs CNO ***p = 0.0002, ketamine vs CNO ns *p* = 0.9971. Data were presented as means ± s.e.m and were analyzed by *t*-test or ANOVA and Tukey’s post hoc test. Each replicate is a different mouse. For IHC, each point represents one mouse, with 3–5 sections averaged per mouse. Source data and complete statistics are provided as a source data file. Scale bar 25 μm.

Finally, we tested whether ABIN activity is not only necessary but also sufficient, for ketamine’s antidepressant effects. To test our hypothesis, this time we used the activating DREADD, hM3Dq, a synthetic receptor coupled to Gq protein to increase cellular activity, which is activated by exogenous ligand CNO^56,58^. We used the Ascl1-Cre;hM3Dq (hM3Dq) mouse line described previously^40^ for specific and inducible activation of ABINs. Two days after TAM injections, hM3Dq mice were exposed to UCMS for 21 days. At this point, three weeks after the last TAM treatment, HA-tagged hM3Dq is expressed almost exclusively in NeuN^+^Calb^–^ ABINs^59^. Mice were then given a single dose of either CNO, ketamine, or vehicle 24 h prior to behavioral testing (Fig. 5a). Mice displayed no locomotor differences among groups (Fig. 5b). ABIN activation by CNO administration induced an antidepressant effect of the same magnitude as ketamine, as measured by the increased preference for social interaction (Fig. 5c) and novelty (Fig. 5d), and decreased time immobile (Fig. 5e).

Additionally, fluorescence staining (Fig. 5f) showed that while neither ketamine nor CNO treatment altered the total number of HA^+^ cells (Fig. 5g), ketamine and CNO both significantly increased the number of activated ABINs (EGR1^+^HA^+^) (*p* < 0.001 and *p* < 0.0001, respectively) (Fig. 5h). The total number of EGR1^+^ cells increased in both the ketamine- and CNO-treated groups (*p* < 0.001, Fig. 3h). In total, these data demonstrate that ABIN activity is sufficient for ketamine’s rapid behavioral effect.

## Discussion

Although ketamine can exert a rapid antidepressant effect, symptoms reemerge as the effects of a single dose diminish, and repeated administration of even low doses can have addictive and deleterious effects^8–10^. To circumvent the limitations of ketamine use, there has been great interest in investigating other drugs and targets to elicit a similar rapid antidepressant response with fewer risks. However, thus far few other drugs have produced effects comparable to the speed and magnitude of ketamine’s^74,75^. Identification of a single cell population in one area of the brain that can be targeted to mimic the antidepressant activity of ketamine can potentially help with the development of drugs that do not produce as many side effects as ketamine, which exerts effects in multiple areas of the brain.

In this study, we selectively modulated the activity of adult-born immature granule neurons (ABINs) in the mouse DG to determine the nature of ABINs’ contribution to the rapid behavioral effects of ketamine. The use of DREADDs (hM4Di and hM3Dq) provided a means to specifically regulate the activity of ABINs while avoiding the potential confounding effects of altering neurogenesis, changing the number of ABINs, compensation in developmental models, or damage to the neurogenic niche. Loss of ABIN activity in the DG blocked ketamine-induced behavioral changes, indicating that ABIN activation is necessary for these changes. Conversely, activation of ABINs was sufficient to induce behavioral effects of the same quality and magnitude as ketamine, indicating that ABIN activity is, by itself, sufficient to produce the behavioral changes seen after ketamine treatment. We also observed that ABIN activity is reduced by NBQX, a known inhibitor of ketamine’s antidepressant effects that acts via blockade of AMPARs^23,24,73^, indicating that AMPAR activity is an upstream signal that activates ABINs. Taken together, these data demonstrate that activation of ABINs in the DG of the hippocampus is both necessary and sufficient for the rapid behavioral effects of ketamine.

In mammals, adult neurogenesis occurs in the hippocampal dentate gyrus and in the subventricular zone, and substantial evidence links dysfunction of the hippocampal neurogenic niche to major depressive disorder (MDD). Hippocampal neurogenesis decreases with MDD and increases with antidepressant treatment^32–34,76^. Increasing neurogenesis can rescue the effects of chronic stress while inhibiting neurogenesis blocks the ameliorative effects of antidepressants^32,35–37^. Ketamine induces numerous effects on the hippocampus, both rapidly (e.g., increased dendritic spines^77^ and reduction of synaptic inhibitory input^78^) and after a delay (e.g., increased neurogenesis^79^). Studies in vitro have shown that direct application of ketamine to hippocampal slices induces synaptic potentiation^20,26,74,80–83^ or pyramidal cell activation^78^, acting directly on hippocampal NMDA receptors. However, in vivo, ketamine’s behavioral effects require alterations in numerous brain regions, suggesting that circuitry beyond the hippocampus is necessary for ketamine’s ultimate behavioral effect. Our work bridges these observations by suggesting how numerous signals may converge on the hippocampus and produce an antidepressant effect. ABINs regulate the circuitry of the DG, specifically by regulating the activity of mature granule neurons according to their presynaptic partners^84^. As reviewed by Doan et al. 2019, ABINs establish glutamatergic synapses onto hilar GABAergic interneurons to inhibit mature granule cells, while weakening inhibitory synaptic currents through a different mechanism^85^. In this manner, alterations in ABIN activity can modulate existing DG circuits to provoke downstream effects. Recent work demonstrates that signaling at hippocampal CA3-CA1 synapses, downstream of the DG, are essential for ketamine-induced long-term potentiation^78,83^, providing another event in this cascade that supports the role of hippocampal circuitry in rapid antidepressant effects. Future work is warranted to examine whether increased ABIN activity leads to changes in CA3-CA1 signaling after ketamine treatment.

The dorsal and ventral hippocampus regulate different behaviors and neuronal circuitry^86–92^. Our work suggests that low-dose ketamine acts predominantly on the ventral hippocampus. This is consistent with the observation that patients given a single administration of low-dose ketamine show some initial negative cognitive effects, but after 24 h, only the affective antidepressant changes persist^71^. Given the association between the dorsal hippocampus and cognition and ventral hippocampus and mood, our results provide a possible explanation for ketamine’s predominantly mood-related effects.

The hippocampus also plays a role in the sustained antidepressant effects of ketamine. After a single dose of ketamine, patients continue to experience antidepressant effects long after the drug has been metabolized and excreted^93^, implicating processes that outlast the presence of the drug. In rodents, NMDAR antagonism increases neurogenesis^94^, and the sustained behavioral effects of ketamine are blocked by ablation of the neurogenic niche^95^, suggesting that the DG contributes to both the acute and the sustained mechanisms of ketamine action. Our data corroborate prior work demonstrating that neurogenesis does not have to be altered for the acute effect of ketamine to be realized, as neural progenitor cells, which have not yet become immature neurons at the time of ketamine treatment, do not contribute to acute effects^41^. Our work demonstrates that altering the activity, but not the number, of ABINs mediates the rapid effects of ketamine. However, the longer-term effects of ketamine may be influenced by changes in neurogenesis.

Our use of the drug NBQX highlights that the ketamine-induced increase in ABIN activity is dependent on AMPAR activity. AMPAR potentiation and positive allosteric modulation also have antidepressant effects, but they do not have the same duration or magnitude as ketamine^96,97^. Additional work is needed to determine the critical site(s) of AMPAR receptor effects-whether upstream of the hippocampus, or even on ABINs themselves. AMPAR activity in different brain regions (e.g., medial prefrontal cortex, mPFC) is necessary for acute ketamine effects and may provide important input to the DG circuitry^20,24,73^. However, ABINs themselves express both NMDARs and AMPARs^53,98,99^, so it is possible that AMPARs in the hippocampus or DG itself are directly mediating some of the effects of ketamine. Retrograde neuronal tracing studies paired with conditional, specific knockouts of the AMPAR may be required to determine which cell populations are responsible for the AMPAR activity that stimulates ABINs.

Activating ABINs using hM3Dq recapitulated ketamine effects on the variety of behaviors tested here. However, behavior and cognition have many facets, and it is possible that there are behavioral or downstream signaling differences between hM3Dq activation and ketamine treatment that we did not see in the assortment of tests that we used. While our testing paradigm was informed by numerous previously published studies and included DG-dependent depression-like behavior tests and anxiety-like behavior tests, which in sum assessed spatial, social, and memory-related behaviors, future work may include a detailed assessment of possible differences in adverse (e.g., addictive and hedonistic) effects between ketamine treatment and ABIN activation, especially because a key risk of ketamine use is addiction potential. Different signaling pathways appear to be involved in ketamine-induced antidepressant behaviors compared to its addictive ones^100^, and tracing studies could also help identify whether cell activation differentially regulates the balance of activity and modification of neuronal circuitry. Prior reports have shown that ketamine alters circuitry between the mPFC and other areas of the brain^101,102^, and in fact, that the activity of a ventral hippocampus to the mPFC pathway underlies ketamine’s acute antidepressant effect^102^. These studies strongly support our conclusions and inform our hypothesis that the increased activity of immature neurons regulates this circuitry by inhibiting mature neurons, which in turn provide altered signals to downstream pathways and areas of signaling.

Taken together, these results establish the necessity of ABIN activity for ketamine’s antidepressant effects and the sufficiency of this activity to induce these effects on the same time scale and magnitude as ketamine, providing a strong basis for future studies focused on targeting ABINs and the pathways that regulate them.

## Methods

### Mouse lines

All animal procedures were approved by the Northwestern University Institutional Animal Care and Use Committee (IACUC). All experiments were performed in accordance with the Public Health Service Policy on Humane Care and Use of Laboratory Animals. All animals were housed 3–5 per cage on a 14:10 h light:dark cycle in a controlled environment and received food and water ad libitum. Mice were always housed within the following limits: acceptable temperature range 64 79 °F (typically 72–74 °F); humidity range: 30–70%. All behavioral testing was conducted during the light period.

For every experiment, we used nearly equal cohorts of male and female mice in each group. All analyses were first conducted to compare behavioral effects by sex (Supplementary Fig. 1a–d). Upon finding no behavioral differences between the sexes for any of our tests, we reported the results for both sexes together. Eight- to 10-week-old, naive C57Bl/6 male and female mice (Charles River, Wilmington, MA, USA) were used for ketamine and NBQX experiments (Figs. 1,  4), where animals were randomly assigned to experimental groups: saline or ketamine treatment (Fig. 1) or vehicle/NBQX or saline/ketamine treatment (Fig. 4). hM4Di and hM3Dq floxed mice (hM4Di−/+—Stock#026219 and hM3Dq−/+—Stock#026220)^56^ were purchased from Jackson Laboratories (Bar Harbor, ME, USA) and were mated to mice containing tamoxifen-inducible Cre recombinase under the control of the Ascl1 promoter (Ascl1-CreER^TM^)^44,59,103^ to produce double-transgenic progeny, Ascl1-CreER^TM^;R26^LSL−hM4Di^ (Ascl1-Cre;hM4Di), and Ascl1-CreER^TM^;R26^LSL−hM3Dq^ (Ascl1-Cre;hM3Dq). Mice were genotyped by PCR with genomic DNA and primer sequences provided by Jackson Laboratories. Tamoxifen-induced expression of hM4Di and hM3Dq was performed at 8–10 weeks of age. Tamoxifen administration led to a conditional expression of HA-tagged hM4Di and hM3Dq in Ascl1-expressing cells. Cre and/or DREADD-negative littermates from heterozygote breeding were used as controls. At least three independent cohorts of mice were run for all experiments.

#### hM4Di mice

The inhibitory DREADD hM4Di is a synthetic muscarinic receptor coupled to the Gi protein^57,58^ and is activated by the synthetic compound Clozapine-N-Oxide (CNO)^57^. The Ascl1-Cre;hM4Di mouse model, herein called “hM4Di,” has been described previously^40^. Briefly, we induced Cre-dependent expression of HA-tagged hM4Di in Ascl1-expressing type-2 transient amplifying neural progenitor cells (NPCs) and their progeny, as well as a small subset of Type 1 Neural Stem Cells (NSCs) by injecting tamoxifen in adult Ascl1-Cre;hM4Di mice^104^. Neural progenitors labeled at the Ascl1^+^ stage become immature neurons in 28 days, with no perturbations in circuit formation in the niche. This approach has been validated in our lab, using both imaging and electrophysiology, as an effective way to manipulate DREADD expression, neuron labeling, and neuronal activity, without notable mouse mortality^40^. CNO is also given by water bottle for 24 h following CNO injection to ensure continued neuronal silencing. One day later, behavioral tests are conducted, and tissue is immediately fixed for immunohistochemistry. Previous work has shown that CNO has no behavioral or electrophysiological effect in Cre- or hM4Di- mice^40^.

#### hM3Dq mice

The excitatory DREADD hM3Dq is a synthetic muscarinic receptor coupled to the Gq protein^57,58^ and similar to hM4Di, it is activated by CNO^57^. The Ascl1-Cre;hM3Dq mouse model, herein called “HM3Dq”, has been described previously^40^. Other than the hM3Dq transgene, this line is the same as the hM4Di mouse line.

### Drug administration

Mice were weighed prior to drug administration. Ketamine (100 mg/mL, Henry Schein NDC# 11695-0702-1) was diluted in saline to a working concentration of 0.6 mg/mL and administered intraperitoneally (i.p.) at a 3 mg/kg dose. NBQX (Tocris #1044) was dissolved in sterile water and was administered via subcutaneous injection at 10 mg/kg. All drugs including saline and water vehicle controls were administered at a volume of 100 uL per 20 g mouse.

For conditional expression of hM4Di and hM3Dq, tamoxifen (Sigma Aldrich) was dissolved in 100% ethanol (10%), suspended in corn oil (90%), and administered via i.p. injection at 120 mg/kg for 5 consecutive days. A total injection volume of 100 uL/ 20 g animal was used.

Water-soluble CNO was purchased from Hello Bio (Princeton, NJ, USA). For hM4Di experiments, animals received a single i.p. injection of 5 mg/kg CNO 26 h before the behavioral tests. Control animals received i.p. vehicle injections in the same time course. In addition, CNO was dissolved in sterile drinking water at a concentration of 0.025 mg/ml according to previous work^40^. At this concentration, the mice received ~5 mg/kg per day CNO^40^. CNO-group animals had this CNO solution as their only source of drinking water ad libitum for 26 h until they were sacrificed for sample collection. Vehicle treatment included regular drinking water. For hM3Dq experiments, a single i.p. injection of 2.5 mg/kg CNO was administered 24 h before the behavioral tests.

### Unpredictable chronic mild stress (UCMS)

Mice were subjected to various unpredictable chronic mild stressors for 3 weeks. Before exposure to any stressor, animals were transferred to a clean room used for unpredictable chronic mild stress (UCMS) manipulations. Mice were exposed to 1 or 2 common stressors each day for a minimum of 2 h, except light cycle disturbances and restraint stress (Supplementary Table 1)^40^. Stressors were performed on a randomized schedule (Supplementary Table 2). At the end of each daily stress period, animals were placed in clean cages and returned to the housing facility. Control, stress-naïve animals were handled only for cage changes, drug injection, and behavioral tests.

### Behavioral testing

For all behavioral analyses, mice were transferred to the testing room 1 h prior to testing for acclimation to the test environment. Every behavioral apparatus was wiped with 70% ethanol prior to each trial and between trials. The social interaction test (SIT) and social novelty test (SNT) were performed at the Northwestern University Behavioral Phenotyping Core Facility. The tail suspension test (TST) was performed at the nearby satellite area of the Kessler lab. Behavioral analyses were performed by an experimenter blind to the experimental condition. All animals were sacrificed immediately after the last behavioral test to collect brain samples.

#### Three-chamber apparatus and tests

Social interaction and social novelty were evaluated on a three-chamber sociability test, where a center chamber separates two identical chambers. The test mouse was first habituated to the three-chamber apparatus for 5 min. Distance traveled during this habituation period was recorded. For the social interaction test (SIT), a wired cup with an unfamiliar mouse and another cup with a small object were introduced into the two identical side chambers. The test animal was allowed to explore the three chambers freely for 10 min. At the end of 10 min, the test mouse was guided to the center chamber while the apparatus was cleaned and wired cups in the side chambers were replaced. The social novelty test (SNT) occurred immediately after; the object-containing cup was replaced with another cup containing an unfamiliar mouse. The test animal was once again allowed to explore the three chambers freely for 10 min. At the end of 10 min, each mouse (test and unfamiliar mice) was returned to their respective home cages. For each set of experiments, the orientation of the two wired cups containing unfamiliar mice or the object was counterbalanced. Notably, the familiar mouse cup during the SNT was always on the opposite side as for the SIT. The movement of the test mouse and time spent interacting with each cup was tracked and recorded with LimeLight 4 software (Actimetrics, Coulbourn Instruments) under white light (250 lux). Video recordings of the test were quantified by an experimenter blinded to experimental conditions. All experimental mice housed in the same cage underwent each test at the same time to avoid possible confounding interactions between tested and untested mice. All unfamiliar mice were of the same age and sex as the test mice. Unfamiliar mice were from different litters than the test mice and had never interacted with them. They were weight-matched within ±1 g of each test mouse.

#### Tail suspension test (TST)

The tape was affixed to the test mouse’s tail 2 cm from the tip and the mouse was suspended from a horizontal bar at a height of 30 cm, and testing was conducted under white light (250 lux). The mouse was suspended for 6 min and video recordings of the test were quantified by an observer blinded to experimental condition. The total time spent in an immobile posture was measured.

#### Open field test (OFT)

The OFT apparatus consisted of a 56 × 56 cm open arena with 30 cm high walls under white light (250 lux). The mouse was placed in the center of the arena and allowed to move freely for 5 min with the activity being recorded and tracked by LimeLight 3 software (Actimetrics, Coulbourn Instruments). The software recorded and analyzed the total distance traveled in the inner (28 × 28 cm central area of the OF) and outer areas of the arena.

### Immunohistochemistry

Mice were transcardially perfused with PBS followed by 4% paraformaldehyde. Brains were fixed overnight in 4% paraformaldehyde and then transferred to 30% sucrose for 24 h. Forty-micrometer-thick floating sections were obtained using a microtome (Microm HM 450, Thermo Fisher, Waltham, MA, USA). Tissue sections were washed three times in PBS, blocked in 10% normal serum with 0.25% Triton X-100 in PBS for 1 h at room temperature (RT), and incubated overnight at 4 °C in primary antibody diluted in 1% bovine serum albumin (BSA) with 0.25% Triton X-100 in PBS. Sections were washed three times in PBS and then incubated for 1 h at RT with a fluorophore-conjugated secondary antibody (Alexa-488, Alexa-555, or Alexa-647, Thermo Fisher) and 4′,6-diamidino-2-phenylindole (DAPI) for nuclear stain (Invitrogen Hoescht 33258, Carlsbad, CA, USA). Following three final washes in PBS, floating sections were mounted with ProLong Gold Antifade Reagent (Life Technologies, Carlsbad, CA, USA).

Primary antibodies: chicken anti-Calbindin (1:1000, #CPCA, EnCor), chicken anti-Calretinin (1:1000, NBP2-50029, Novus), rabbit anti-c-Fos (1:500, #26192–1-AP, Proteintech), guinea pig anti-Doublecortin (1:500, AB2253, Millipore), rabbit anti-EGR1 (1:500, #4153, Cell Signaling), rat anti-HA-Tag (1:250, # 3F10, Roche), mouse anti-NeuN (1:500, MAB377, Millipore).

Fluorophore-conjugated secondary antibodies (1:250, Thermo Fisher): Alexa-488 (goat anti-rabbit A11034, goat anti-chicken A-11039, goat anti-rat a11006, goat anti-guinea pig A-11073), Alexa-555 (goat anti-rabbit A-21428, goat anti-mouse A21127), Alexa-647 (goat anti-rat A-21247, goat anti-chicken A21449, goat anti-mouse A21240), and 4,6-diamidino-2-phenylindole (DAPI) was used at 1:1000 for nuclear stain (Invitrogen Hoescht 33258, Carlsbad, CA, USA).

### Confocal imaging and quantification

Images were acquired using a Leica TCS SP5 Confocal Microscope. Z-stacks of the dentate gyrus were obtained (step size: 3 µm) using sequential scanning to prevent bleed-through between fluorophores. Six or more Z-stacks of equal thickness and equivalent rostrocaudal position were quantified for each sample. Dorsal hippocampal sections were defined by bregma greater than −2.3 mm; ventral hippocampal sections were defined by bregma less than −2.8 mm^91,92,105^. Stereological cell counting was performed using ImageJ software. For per volume quantification, cell counts were normalized to the volume of the dentate gyrus granule cell layer. All imaging and quantification were performed blinded to experimental conditions.

### Statistical analysis

Statistical analyses were performed via two-tailed Student’s *t*-test, two-way ANOVA, or three-way ANOVA with Tukey’s post hoc comparison test for correction for multiple comparisons, as indicated in the figures and figure legends. GraphPad Prism 9 software was used for the analyses. Normality was assessed using the Shapiro–Wilk and Kolmogorov–Smirnov tests and equality of variances was verified using the *F*-test of equality of variance. The significance threshold used was *p* < 0.05. The exact sample size for each experimental condition is represented in the figures as symbols. All data were reported as means ± s.e.m. Complete statistical data for each experiment are provided in the Source Data file.

### Reporting summary

Further information on research design is available in the Nature Research Reporting Summary linked to this article.

## Supplementary information

Supplementary Information

Reporting Summary

## Source data

Source Data

## Data Availability

All data needed to evaluate the conclusions in the paper are present in the paper and/or the Supplementary Materials. The datasets generated and analyzed during the current study, and all additional related data, are available from the corresponding author on request. Source data are provided with this paper.
