# Author Correction: Ketamine activates adult-born immature granule neurons to rapidly alleviate depression-like behaviors in mice

**DOI:** 10.1038/s41467-025-66663-2

**Published:** 2025-11-21

**Authors:** Radhika Rawat, Elif Tunc-Ozcan, Tammy L. McGuire, Chian-Yu Peng, John A. Kessler

**Affiliations:** Department of Neurology, Feinberg School of Medicine, Northwestern University, Chicago, IL 60611 USA

**Keywords:** Adult neurogenesis, Depression, Cellular neuroscience, Depression

Correction to: *Nature Communications* 10.1038/s41467-022-30386-5, published online 12 May 2022

In the version of the article initially published, in the “Drug administration” section of the Methods, the text “CNO was dissolved in sterile drinking water at a concentration of 0.25 mg/ml” should have read “CNO was dissolved in sterile drinking water at a concentration of 0.025 mg/ml” and has now been corrected in the HTML and PDF versions of the article.

