# Supplementary material for: Ketamine activates adult-born immature granule neurons to rapidly alleviate depression-like behaviors in mice
Source: Nat Commun. 2022 May 12;13:2650. doi: 10.1038/s41467-022-30386-5 (PMC9098911; doi:10.1038/s41467-022-30386-5)

Supplementary Table 1: Stressors used in the Unpredictable Chronic Mild Stress (UCMS) paradigm

Stressor	Description	Duration
Wet bedding	Home cage bedding was dampened by pouring ~500 ml of clean water, which would be enough for not to cause pooling of water.	2h
No bedding	All bedding from each home cage was removed.	2h
Tilted cage	Home cage was tilted ~45° by using a sturdy object that would remain in place as the animal moves around.	2h
Light cycle disturbances	Animals were exposed to regular room light during the night period or regular room light was off during day time.	24h
Social stress	Mice were transferred from their home cage to the cage of a neighboring mice that have been removed for 3 h.	2h
No bedding + water	All bedding from each home cage was removed and water (warmer than room temperature ~30 °C) was added a depth of 5 mm. Animals were towel dried prior to placement into clean cages.	2h
Restraint stress	Mice were placed in a 50-ml plastic Falcon tube with openings in both sides for breathing .	1h
Predator smell	A filter paper soaked with 5 µl of 10% 2,4,5-Trimethylthiazoline (a component of fox feces and the most commonly used synthetic reagent for inducing innate fear in rodents) was placed into the home cage.	2h
No bedding + tilted cage	After removing the bedding, each home cage was tilted ~45° by using a sturdy object that would remain in place as the animal moves around.	2h

Supplementary Table 2: Schedule of Unpredictable Chronic Mild Stress (UCMS) stresses

	**Day 1**	**Day 2**	**Day 3**	**Day 4**	**Day 5**	**Day 6**	**Day 7**
**Week 1**	Restraint stress	No bedding	Predator smells	Tilted cage	No bedding+tilted cage	Predator smells	Light cycle disturbance
	Wet bedding	Tilted cage	No bedding+water	Social stress	Wet bedding		
**Week 2**	No bedding+water	Tilted cage	Predator smells	No bedding	Social stress	Social stress	Restraint stress
	Predator smells	Social stress	Wet bedding	No bedding+tilted cage	Wet bedding		
**Week 3**	Tilted cage	Social stress	Predator smells	Social stress	No bedding	Restraint stress	Light cycle disturbance
	Wet bedding	No bedding+water	No bedding+tilted cage	Wet bedding	No bedding+tilted cage		

Supplementary Figure 1

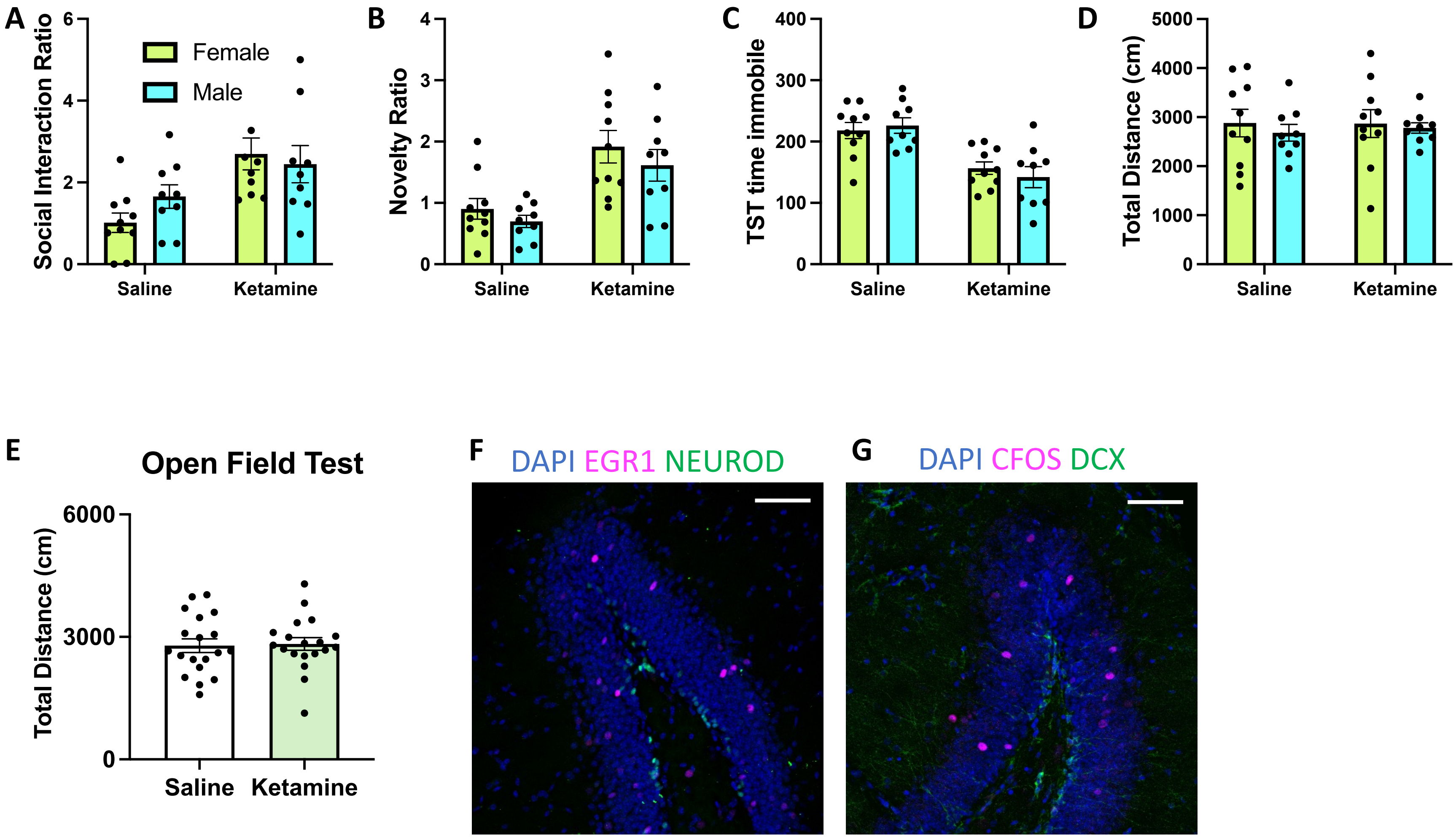

Supplementary Figure 2

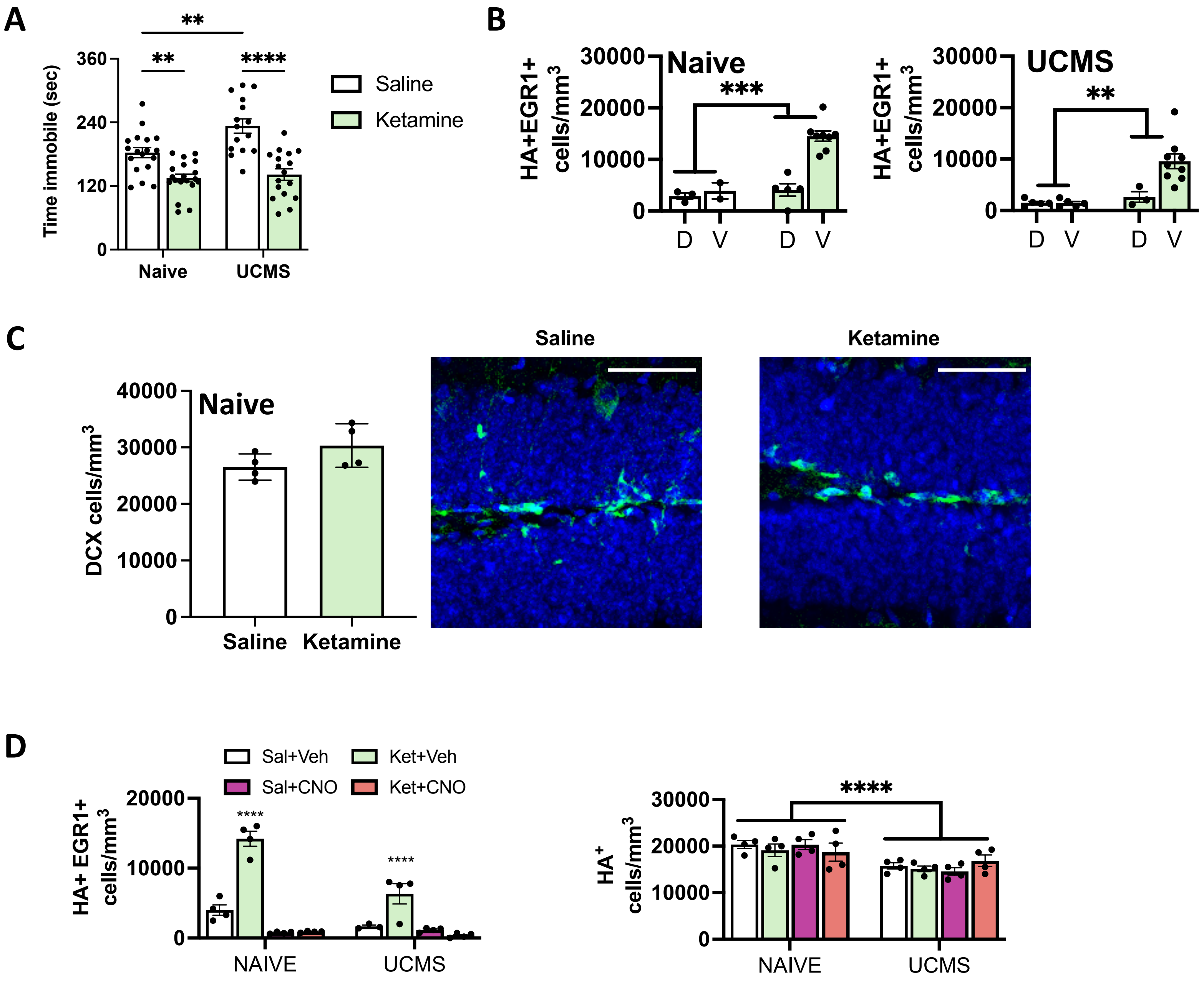

Supplement: Supplementary file 1 — Supplementary Information [file 41467_2022_30386_MOESM1_ESM.pdf]
