# Supplementary material for: Ketamine activates adult-born immature granule neurons to rapidly alleviate depression-like behaviors in mice
Source: Nat Commun. 2022 May 12;13:2650. doi: 10.1038/s41467-022-30386-5 (PMC9098911; doi:10.1038/s41467-022-30386-5)
Supplement: Supplementary file 2 — Reporting Summary [file 41467_2022_30386_MOESM2_ESM.pdf]

nature research

Corresponding author(s): Radhika Rawat

Reporting Summary

Nature Research wishes to improve the reproducibility of the work that we publish. This form provides structure for consistency and transparency in reporting. For further information on Nature Research policies, see Authors & Referees and the Editorial Policy Checklist.

Statistical parameters

When statistical analyses are reported, confirm that the following items are present in the relevant location (e.g. figure legend, table legend, main text, or Methods section).

n/a	Confirmed	
☐	☒	The exact sample size (n) for each experimental group/condition, given as a discrete number and unit of measurement
☐	☒	An indication of whether measurements were taken from distinct samples or whether the same sample was measured repeatedly
☐	☒	The statistical test(s) used AND whether they are one- or two-sided<br>*Only common tests should be described solely by name; describe more complex techniques in the Methods section.*
☐	☒	A description of all covariates tested
☐	☒	A description of any assumptions or corrections, such as tests of normality and adjustment for multiple comparisons
☐	☒	A full description of the statistics including central tendency (e.g. means) or other basic estimates (e.g. regression coefficient) AND variation (e.g. standard deviation) or associated estimates of uncertainty (e.g. confidence intervals)
☐	☒	For null hypothesis testing, the test statistic (e.g. F, t, r) with confidence intervals, effect sizes, degrees of freedom and P value noted<br>*Give P values as exact values whenever suitable.*
☒	☐	For Bayesian analysis, information on the choice of priors and Markov chain Monte Carlo settings
☒	☐	For hierarchical and complex designs, identification of the appropriate level for tests and full reporting of outcomes
☒	☐	Estimates of effect sizes (e.g. Cohen's d, Pearson's r), indicating how they were calculated
☐	☒	Clearly defined error bars<br>*State explicitly what error bars represent (e.g. SD, SE, CI)*

Our web collection on statistics for biologists may be useful.

Software and code

Policy information about availability of computer code

Data collection	LimeLight 3 and LimeLight 4 software (Actimetrics, Coulbourn Instruments, USA) was used to collect and analyze the data for the time spent interacting with objects and mice in the Social Interaction and Social Novelty Tests and total distance traveled in the open field test. Total immobility in the tail suspension test was manually scored via video recordings by an experimenter blind to genotype and experimental condition. Stereological cell counting was performed using ImageJ software (version 1.53a). All imaging and quantification were performed blinded to mouse genotype and experimental condition.
Data analysis	GraphPad Prism 9 was used for the data analyses.

For manuscripts utilizing custom algorithms or software that are central to the research but not yet described in published literature, software must be made available to editors/reviewers upon request. We strongly encourage code deposition in a community repository (e.g. GitHub). See the Nature Research guidelines for submitting code & software for further information.

Data

Policy information about availability of data

All manuscripts must include a data availability statement. This statement should provide the following information, where applicable:

- Accession codes, unique identifiers, or web links for publicly available datasets
- A list of figures that have associated raw data
- A description of any restrictions on data availability

All data needed to evaluate the conclusions in the paper are present in the paper and/or the Supplementary Materials. The data generated in this study are provided in the Source Data file.

Field-specific reporting

Please select the best fit for your research. If you are not sure, read the appropriate sections before making your selection.

☒ Life sciences ☐ Behavioural & social sciences ☐ Ecological, evolutionary & environmental sciences

For a reference copy of the document with all sections, see nature.com/authors/policies/ReportingSummary-flat.pdf

Life sciences study design

All studies must disclose on these points even when the disclosure is negative.

Sample size	The number of animals in each group necessary for each measurement was determined by a power analysis done with G*Power 3.1.9.2 using the standard deviations and effect sizes from our preliminary studies, an alpha of 0.05, and a power (1-b) of 0.80. Experiments were run with sufficient numbers to examine possible sex-differences.
Data exclusions	Exclusion criteria were both experimental and statistical. Exclusion occurred for mice that escaped the testing area during the three-chamber test (4 mice) and mice that were not securely suspended during the tail suspension test (5 mice). One animal was excluded due to unrelated tooth overgrowth and concomitant weight loss. One cage (4 mice) was excluded due to environmental stress exerted on the stress-naive group. Exclusion also occurred for mice that traveled a total distance that was identified as a statistical outlier in either direction (determined by the standard ROUT analysis in GraphPad Prism 9 software). The mice excluded from the study were not associated with any individual experimental group.
Replication	All attempts at replication were successful. Main effects were consistent across at least three different cohorts for all experiments.
Randomization	For all behavioral studies, mice were randomly assigned to experimental groups by sampling across different litters to prevent litter effects. For staining (immunohistochemistry), subsets of each condition were selected at random.
Blinding	Investigators were blinded to the experimental groups until all data had been collected and analyzed.

Reporting for specific materials, systems and methods

Materials & experimental systems

n/a	Involved in the study
☒	☐ Unique biological materials
☐	☒ Antibodies
☒	☐ Eukaryotic cell lines
☒	☐ Palaeontology
☐	☒ Animals and other organisms
☒	☐ Human research participants

Methods

n/a	Involved in the study
☒	☐ ChIP-seq
☒	☐ Flow cytometry
☒	☐ MRI-based neuroimaging

Antibodies

Antibodies used	Primary antibodies: chicken anti-Calbindin (1:1000, #CPCA, EnCor), guinea pig anti-calretinin (1:1000, CRgp7, Swant), rabbit anti-c-Fos (1:500, #26192–1-AP, Proteintech), guinea pig anti-Doublecortin (1:500, AB2253, Millipore), rabbit anti-EGR1 (1:500, #4153, Cell Signaling), rat anti-HA-Tag (1:250, # 3F10, Roche), mouse anti-NeuN (1:500, MAB377, Millipore).<br><br>Fluorophore-conjugated secondary antibodies (1:250, Thermo Fisher): Alexa-488 (goat anti-rabbit A11034, goat anti-chicken

A-11039, goat anti-rat a11006, goat anti-guinea pig A-11073), Alexa-555 (goat anti-rabbit A-21428, goat anti-mouse A21127), Alexa-647 (goat anti-rat A-21247, goat anti-chicken A21449, goat anti-mouse A21240), and 4,6-diamidino-2-phenylindole (DAPI) was used at 1:1000 for nuclear stain (Invitrogen Hoescht 33258, Carlsbad, CA, USA).

Validation

Each antibody was validated for the species and application with information available from the manufacturer's website.

Animals and other organisms

Policy information about studies involving animals; ARRIVE guidelines recommended for reporting animal research

Laboratory animals

Naïve C57Bl/6 male and female mice (Charles River, Wilmington, MA, USA) were used for antidepressant experiments. hM4Di and hM3Dq floxed mice (hM4Di-/+ - Stock#026219 and hM3Dq-/+- Stock#026220) were purchased from Jackson Laboratories (Bar Harbor, ME, USA) and mated with Ascl1-CreERTM mice to produce double transgenic mice; Ascl1-CreERTM;R26LSL-hM4Di and Ascl1-CreERTM;R26LSL-hM3Dq. Experiments were started when the mice reached the age of eight to ten-weeks, and both male and female mice were used in all experiments.
Mice were housed within the following limits at all times: acceptable temperature range 64-79 degrees Fahrenheit (ideal 72-74F); acceptable humidity range: 30-70%.

Wild animals

This study did not involve wild animals.

Field-collected samples

This study did not involve samples collected from the field.
